# Expression of Vascular Endothelial Growth Factor (VEGF) in Colorectal Adenoma and Carcinoma in a Tertiary Care Center

**DOI:** 10.7759/cureus.31393

**Published:** 2022-11-11

**Authors:** Subalakshmi Balasubramanian, Nagarajan Priyathersini, Thanka Johnson

**Affiliations:** 1 Pathology, Sri Ramachandra Institute of Higher Education and Research, Chennai, IND; 2 Pathology, Sree Balaji Medical College and Hospital, Chennai, IND

**Keywords:** colorectal cancer, adenocarcinoma, adenoma, vegf, angiogenesis

## Abstract

Introduction: Colorectal cancer is one of the malignancies in which angiogenesis has been implicated and its importance at different stages of malignant disease. Neovascularization begins when the angiogenic switch is turned on and when angiogenesis activators outweigh angiogenic inhibitors. The process of blood vessel formation is regulated through several growth factor systems. The vascular endothelial growth factor (VEGF) system is one of the important ones and may consequently be a key system in relation to different aspects of colorectal carcinoma treatment.^ ^Because of the well-defined steps in its progression (adenoma - Tis - T1 invasive cancer- T2 advanced cancer with metastases), colorectal cancer (CRC) represents a model for investigating the effects of angiogenesis throughout tumor development.^ ^ The aim of this study is to determine the role of VEGF expression by immunohistochemistry (IHC) in normal epithelium, dysplasia, carcinoma of colorectal specimens and to correlate the same with tumor grade, stage, nodal status, and metastasis.

Methods: This is a retrospective study on paraffin blocks of 50 colon cancer specimens, 40 adenoma specimens, and 10 normal colonic mucosa specimens. Immunohistochemical stain for VEGF was done on the sections along with controls. Monoclonal antibody detected against VEGF antigen was observed in the cytoplasm of the tumor cells and the intensity of VEGF expression in individual tumor cells was scored on a scale of 0 (no staining) to 3 (strong intensity), and the percentage of cells with VEGF staining at each intensity was estimated from 0 to 100. Pearson’s Chi-squared test and Mann-Whitney test were used to determine significant clinicopathological differences between VEGF expression in positive and negative tumors.

Results: In normal epithelium, VEGF immunoreactivity was seen in all 10 cases with high intensity. Among adenomas, VEGF expression was seen in 26 (65%) of the 40 cases. Out of which in tubular adenomas VEGF expression was seen in 13 cases (60%) and negative in eight cases (40%). In tubulo villous adenoma, VEGF expression was seen in nine cases (60%) and negative in six cases (40%). Villous adenomas showed VEGF expression in all four cases (100%). In adenocarcinoma, VEGF expression was seen to be expressed in 42 cases (84%) and negative in eight cases (16%) and expression was higher in low-grade carcinomas (70%) compared to high-grade carcinomas. A significant difference in the expression of VEGF among adenomas and carcinomas was observed with higher intensity present in adenoma when compared to carcinoma.

Conclusion: Expression of VEGF could be considered as an early carcinogenic factor in colorectal carcinomas as it is expressed in higher intensity in the precancerous lesion and low-grade and stage 1 adenocarcinoma. Hence, we infer that early colorectal carcinomas are an important model for targeted therapy with antiangiogenic factors for VEGF.

## Introduction

Colorectal cancer (CRC) is the third most common cancer occurring worldwide with an estimation of one million new cases diagnosed each year [1]. In recent years, advances in understanding tumor biology have led to the development of targeted therapies making progress in the treatment of CRC. The most significant and independent prognostic factors accepted to date in CRC remain the tumor node metastasis (TNM) stage. Colorectal cancer is one of the many malignancies in which angiogenesis has been implicated. Angiogenesis is the formation of new capillaries, from either endothelial progenitor cells or from pre-existing vasculature. It is of importance at different stages of malignant disease. VEGF is an important angiogenic factor in primary and metastatic human CRC. VEGF expression has been correlated with increased microvessel count in colon tumors and has been associated with poor outcomes as measured by tumor progression, metastasis, and patient survival. It is a highly specific mitogen for vascular endothelial cells. It was first discovered by Napoleone Ferrara in the year 1989. In 1993, Ferrara reported that inhibition of VEGF-induced angiogenesis by specific monoclonal antibodies resulted in dramatic suppression of the growth of a variety of tumors in vivo. These findings provided important evidence that inhibition of angiogenesis may suppress tumor growth and blocking. In recent decades, a variety of signaling molecules, such as VEGFs and vascular endothelial growth factors receptors (VEGFRs), ephrin-eph receptors, angiopoietin-tie, and the delta-notch system, have been identified as playing important roles in angiogenesis [2]. Among these VEGFs and VEGFRs regulate both vasculogenesis (the development of blood vessels from precursor cells during early embryogenesis) and angiogenesis (the formation of blood vessels from pre-existing vessels at a later stage). Recent studies have found that VEGF is expressed early in the progression of CRCs and VEGF expression has been correlated with increased microvessel count in colon tumors, and have been associated with poor outcomes, as measured by tumor progression, metastasis, and patient survival [3]. In addition to evidence suggesting that VEGF is a prognostic factor in CRC, studies have shown that VEGF may predict response to conventional systemic therapy or local radiotherapy. Hence this study has been designed to determine the expression of VEGF in colonic adenoma and colonic carcinoma by immunohistochemistry and to apply this knowledge in predicting the prognosis of angiogenesis.

## Materials and methods

This is a retrospective study on 100 paraffin blocks of 50 colon cancer specimens, 40 adenoma specimens, and 10 normal colonic mucosa specimens received in the Department of Pathology at Sri Ramachandra University over a period of three years. Immunohistochemical stain for VEGF was done on the sections along with controls.

Permission from the Institutional Ethics Committee was obtained prior to commencing the study (IEC NO: CSP-MED/13/JUN/07/34). Gross findings were recorded and clinical data were obtained from the medical record section and available local area computer network in service. Paraffin blocks made after routine processing of resected colectomy specimens were used for the same. Blocks with sections containing tumors, adenomas, and normal colonic mucosa were chosen for the immunohistochemistry (IHC) study. This IHC study was conducted on an estimated sample size of 50 cases of histopathology-proven colonic carcinoma, 40 adenomas, and 10 normal colonic mucosa.

Hematoxylin and eosin (H&E) stained sections were used to grade colon cancer by architectural and cytological features. Other histopathological features observed were adjacent dysplasia and adenomas. Immunohistochemical staining for VEGF was performed on all 100 cases. Monoclonal antibody detected against VEGF antigen was observed as brown color in the cytoplasm of the tumor cells and the intensity of VEGF expression in individual tumor cells was scored on a scale of 0 (no staining) to 3 (strong intensity) (Table 1 ) and the percentage of cells with VEGF staining at each intensity was estimated from 0 to 100.

**Table 1:** Intensity score of VEGF on the tumor cells. VEGF, vascular endothelial growth factor

Intensity score	Positivity
0	Negative
1+	Weak
2+	Moderate
3+	Intense

The absolute value of the proportion of cells at each intensity level was multiplied by the corresponding intensity value, and these products were added to obtain an immunostaining score ranging from 0 to 300 [4-5].

**Figure 1:**
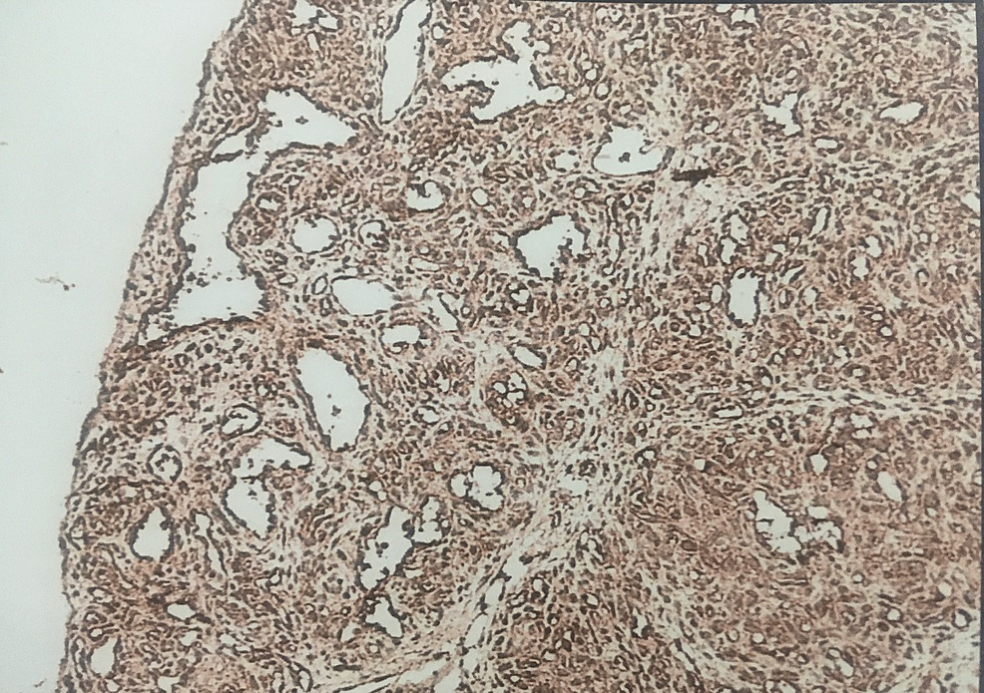
VEGF immunohistochemistry (400x) control -- capillary hemangioma. Normal endothelial cells showing 3+ positivity. VEGF, vascular endothelial growth factor

The previous study showed that the endothelial cells of vessels have strong positivity with VEGF and this was interpreted as a positive control [6]. In our study control was taken from capillary hemangioma (Figure 1) [6]. As negative controls, the slide was treated by replacement of the primary antibody with non-immune serum. 

Statistical analysis

Statistical analysis was done for the data collected by using the “SPSS Version 11” statistical program (IBM Inc., Armonk, NY). Pearson’s Chi-squared test and Mann-Whitney test were used to determine significant clinicopathological differences between VEGF expression in positive and negative tumors. Differences were considered statistically significant when p value was <0.05.

## Results

We studied holoprosencephaly (HPE) proven cases of 50 colorectal carcinomas, 40 colonic adenomas which included 21 tubular adenomas, 15 tubulovillous adenomas, and 4 villous adenomas diagnosed in the Department of Pathology, Sri Ramachandra Medical College and Research Institute over a period of three years. We included 10 normal colonic tissues away from the lesion from the CRC specimens.

In our study of colon cancers, the age of the patients ranged from 27 to 85 years. The highest incidence was noted in the fifth decade. In adenomas, the highest incidence was noted in the sixth decade of life. We found that colon cancer occurred predominantly in men as compared to women with a male-to-female ratio of 3.1 : 1 and in adenomas also males were affected predominantly than females with a ratio of 2.3:1. Rectum was the most common site involved in both colonic cancer as well as in adenomas (32%, 25%). The size of the CRCs ranged from 2.5 to 12 cm out of which up to 5 cm was 48% and more than 5 cm was 52%. In adenomas size ranged from 0.2 to 8 cm out of which in tubular adenomas size ranged from 0.2 to 2 cm and villous adenoma was slightly bigger in size and ranged from 0.3 to 8 cm. There is no definite predilection for large tumors. 

In CRCs, grade 1 was categorized as well differentiated, grade 2 as moderately differentiated, and grade 3 as poorly differentiated. In this study grade 2 is more common (69%) followed by grade 1 (18%).

In our study most of the tumors (50%) belong to pT3 (tumor invades through the muscularis propria into pericolorectal tissues) of the TNM (primary tumor, regional lymph nodes, and distant metastasis) classification, followed by pT2 - (44%) (tumor invades muscularis propria). Regional lymph node metastasis was seen in 48% with N1 - 36% (metastasis in one to three regional lymph node) and N2 - 12% (metastasis in more than four lymph nodes). Nodal metastasis was absent in 52%. Clinically no distant metastasis (cM0) was noted in all the cases except for one case. When the comparison was made for stage, stages 1 and 3 predominated over stages 2 and 4. The high stage was noted predominantly in males and more common in the age group of >60 years.

We analyzed the expression of VEGF in normal colonic epithelium, tubular adenoma, tubulovillous adenoma, villous adenoma, and carcinoma. We noted that the staining of VEGF immunohistochemical stain had taken up in the cytoplasm of all cases (normal colonic mucosa, adenomas, colorectal carcinomas).

**Figure 2:**
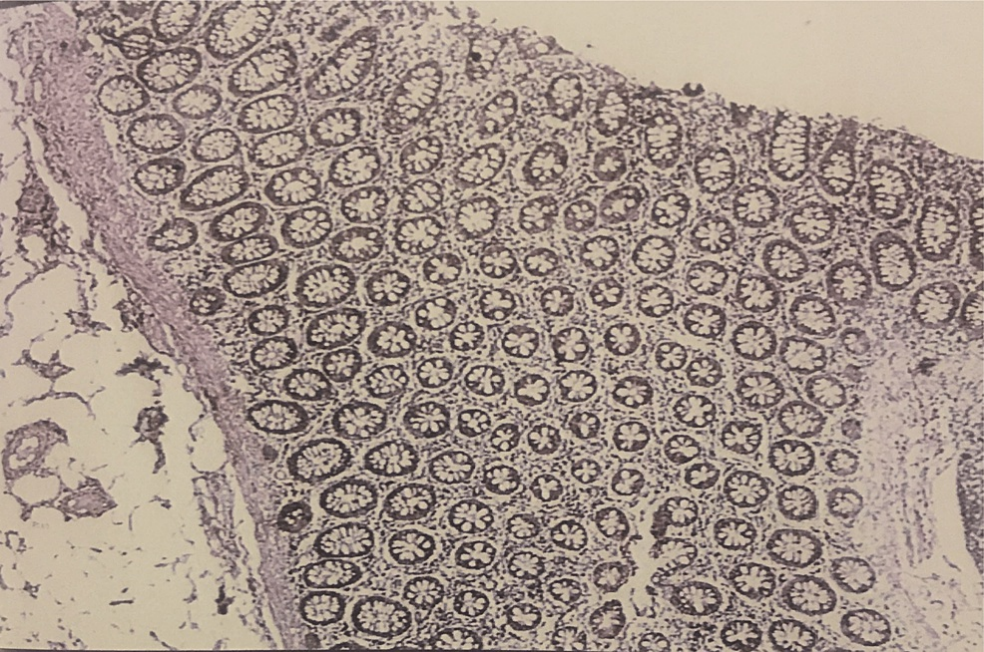
VEGF Immunohistochemistry (400x). 3+ staining pattern in normal colonic mucosa. VEGF, vascular endothelial growth factor

In normal epithelium, VEGF immunoreactivity was seen in all 10 cases with a high intensity of 3+ (Figure 2).

**Figure 3:**
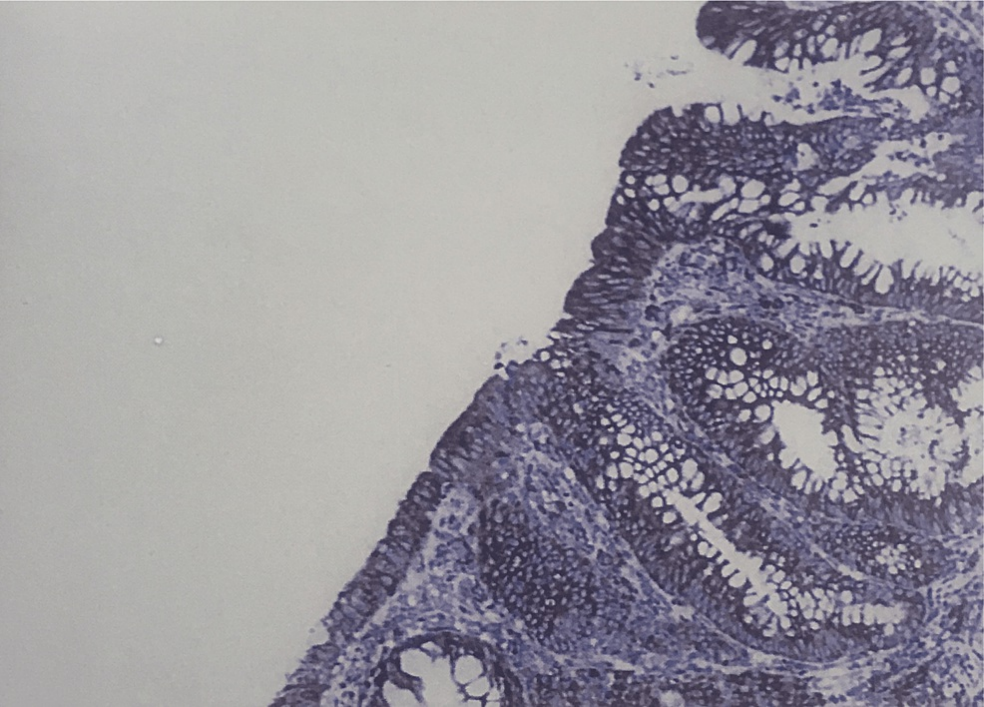
VEGF immunohistochemistry (400x). 3+ staining pattern in tubular adenoma. VEGF, vascular endothelial growth factor

Among adenomas, VEGF expression was seen in 26 (65%) of the 40 cases with an intensity of 2+ in 15 cases and 3+ in 11 cases. In tubular adenoma, 13 cases showed positivity with an intensity of 2+ in nine cases and 3+ in four cases (Figure 3).

**Figure 4:**
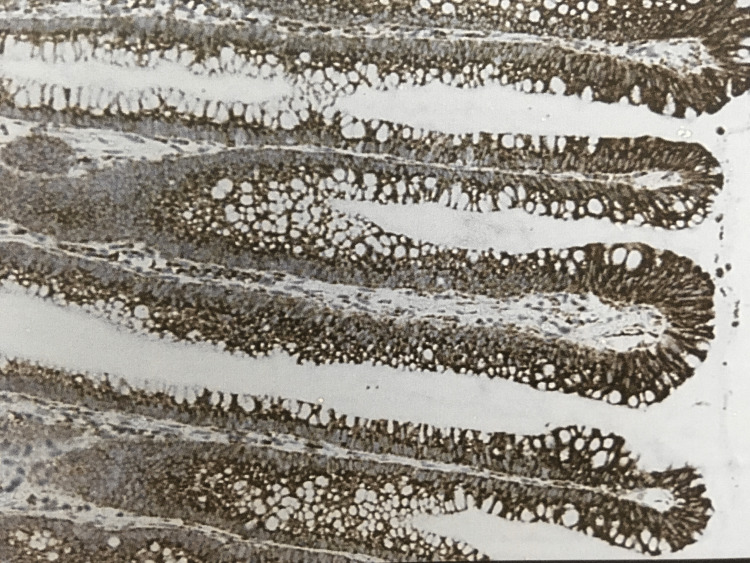
VEGF immunohistochemistry (400x). 3+ staining pattern in villous adenoma. VEGF, vascular endothelial growth factor

In tubulovillous adenoma intensity of 2+ was seen in six cases and 3+ in three cases. In villous adenoma, all four cases showed 3+ positivity (Figure 4).

**Figure 5:**
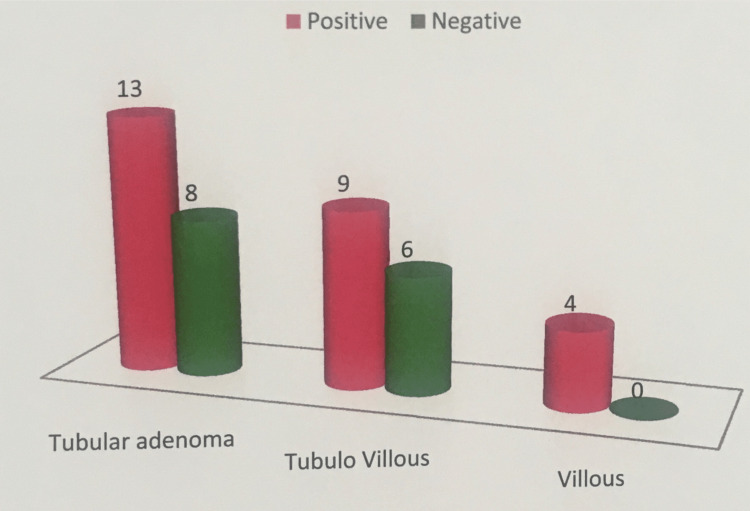
Graphical representation of VEGF expression in adenomas. VEGF, vascular endothelial growth factor

The graphical representation of VEGF expression in tubular adenoma, tubulo villous adenoma, and villous adenoma is depicted in Figure 5. 

In our study we found that there is a significant difference between villous adenoma and tubular adenoma (p= 0.05). The H score ranged from 40 to 300 in villous adenomas and 70 to 300 in tubular adenomas (Table 2).

**Table 2:** Comparison of tubular and villous adenoma with VEGF expression. VEGF, vascular endothelial growth factor

Parameter	No of cases	Median	Score	p Value
Tubular	21	262	70-300	
Tubulo villous + villous	19	180	40-300	0.05

According to the age, VEGF was expressed high in the age group of 61-70 in all the adenomas and there is no significant difference in the expression of VEGF according to the site.

In colorectal carcinomas, VEGF expression when compared based on the age of the patients, showed positivity in 18 cases (58.1%) among the age group <45 years and positivity in eight cases (42.1%) among patients >45 years of age. Thus there is no significant difference with VEGF expression and age of the patients (p = 0.273). When VEGF expression was compared to the size of the tumor, 11 cases (42.3%) were positive in tumors of size up to 5 cm and 15 cases (62.3%) were positive in tumors >5 cm with a p-value of 0.153 which was statistically insignificant.

The clinicopathological data of VEGF expression is given in Table 3. There is no significant difference in VEGF expression on age, sex, and tumor size of the patients.

**Table 3:** Correlation of VEGF expression and positivity with clinicopathological data. VEGF, vascular endothelial growth factor

S. No	Variables	Parameters	Positive	Negative	Total	p Value
1.	Age	>45	18	13	31	0.273
		<45	8	11	19	
2.	Sex	Males	19	19	38	0.324
		Females	8	4	12	
3.	Tumor size	<5 cm	11	15	26	0.153
		>5 cm	15	9	24	

The VEGF expression was analyzed with grade and TNM staging in colorectal carcinomas; the data are summarized in Table 4.

**Table 4:** Correlation of VEGF expression and H score with existing clinicopathological data. VEGF, vascular endothelial growth factor; TNM, tumor, node, and metastase

S. No	Variables		No of cases	Median	Range	p Value
1.	Grade	I	10	260	30-290	0.850
		II	38	210	10-300	
		III	2	180	90-270	
2.	(pT)	1	0	0	0	0.327
		2	22	260	10-300	
		3	25	200	30-300	
		4	3	265	10-300	
3.	Lymph node	N0	0	0	0	0.680
	(pN)	N1	18	260	10-300	
		N2	6	240	20-300	
4.	TNM stage	I	20	265	10-300	0.386
		II	9	200	100-300	
		III	20	130	30-300	
		IV	1	280	280	

**Figure 6:**
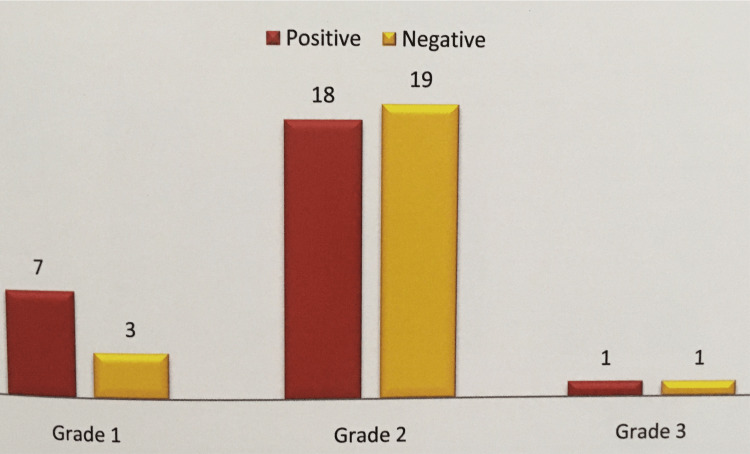
Correlation of VEGF expression with grade in colorectal carcinoma. VEGF, vascular endothelial growth factor

**Figure 7:**
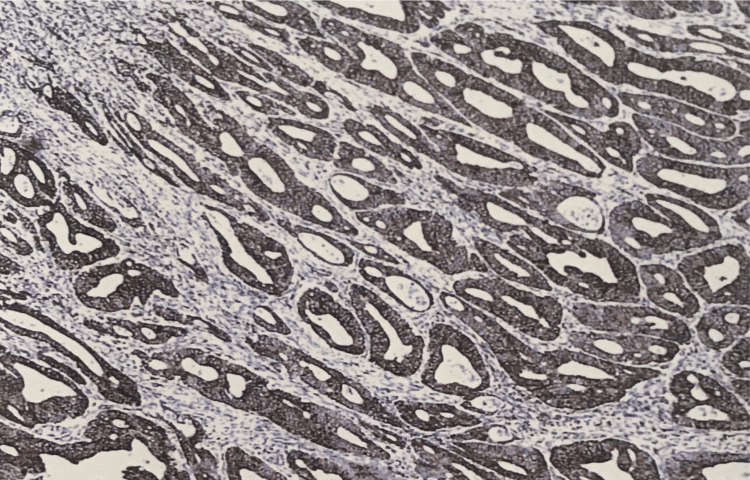
VEGF (immunohistochemistry) 3+ intensity staining pattern in adenocarcinoma. VEGF, vascular endothelial growth factor

In our study, expression of VEGF was seen positive in 7/10 (70%) cases in grade 1 with an intensity of 3+ and showed almost equal positivity in grades 2 and 3 with an intensity of 2+ (47% and 52%) (p=0.443) (Figures 6-7).

**Figure 8:**
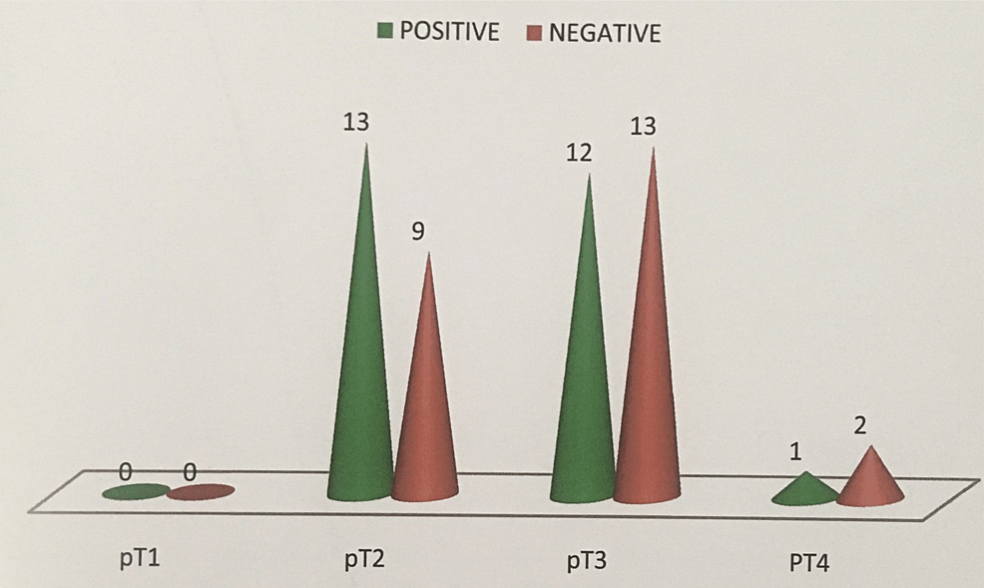
Correlation of VEGF expression with pT stage in CRC. VEGF, vascular endothelial growth factor; CRC, colorectal carcinoma

Expression of VEGF was also analyzed for pT staging, 13 cases of pT2 stage tumors were positive with an intensity of 3+ in eight cases and 2+ in five cases. Some 12 cases of pT3 were positive with an intensity of 2+ in eight cases and 3+ in four cases and out of three cases in pT4 only one case was positive which showed an intensity of 2+ (p=0.600) (Figure 8).

In nodal metastasis, VEGF was positive in eight (44.8%) cases/18 cases in N1 and three (50%)/six cases in N2 were positive with variable intensity and a p-value of 0.684, which was not significant. 

When the expression of VEGF and stage were compared, stage 1 showed maximum expression in 13 cases (65%), H scores ranging from 10 to 300, stage 2 showed expression in four cases (44.4%) H score ranging from 100 to 300, stage 3 (40%) in eight cases score ranging from 30 to 300, and stage 4 in one case (100%) with a score of 280 (p=0.303). In our study, the p-value was higher so there was no correlation with the grade and stage of the tumor (Table 4).

 When the expression of VEGF in adenomas and carcinomas was compared, adenoma had a higher VEGF expression than carcinoma (mean = 216 in adenoma and 195 in carcinoma) (Table 5).

**Table 5:** Comparison of VEGF expression with colorectal adenoma and carcinoma. VEGF, vascular endothelial growth factor

Parameter	N	Mean	Standard deviation	Standard error mean
Carcinoma	50	195.4000	96.59953	13.66124
Adenoma	40	216.2821	72.32709	11.58160

## Discussion

Colorectal carcinoma is the third most common cancer and fourth most frequent cause of cancer death worldwide. The overall five-year survival rate in the United States exceeds 60%, but it is less than 40% in developing countries [7]. The main prognostic factors in volorectal cancers are the stage and grade of the disease. However, these prognostic factors do not fully predict individual clinical outcomes, especially among stage 2 and stage 3 patients. So new prognostic markers are essential to determine patient outcome.

Angiogenesis is the formation of new blood vessels. Tumors cannot grow beyond 1-2 mm in diameter without the development of vascular supply. This is regulated by a delicate balance between local pro-angiogenic and anti-angiogenic factors which are released by both tumor and host cells including endothelial cells and immune system cells. Vascular endothelial growth factor is unique among angiogenic growth factors as it disrupts endothelial barrier function. It is the prototypical pro-angiogenic molecule that has been implicated in several steps throughout angiogenic process [8]. Hence, this capacity might contribute to tumor cell extravasation and metastasis.

More than 90% of the cancers of the colorectal region are adenocarcinomas. Males are affected more often than females. The incidence peaks at 60-70 years of age and fewer than 20% cases occur before 50 years of age [9]. In this study the age group for colorectal carcinoma ranged from 27 to 84 years and peak incidence was at 51-60 years with a male preponderance correlating with worldwide incidence.

Rectal carcinomas are more common in Asians [10]. In this study rectum was the most common site for colorectal adenoma and carcinoma in all the age groups.

Studies have shown that tumor size is of limited prognostic significance [11]. In our study we observed that tumors of varying sizes fell under the same stage because the tumor extent is more important in staging and prognosis than the size.

In general, the degree of gland formation is widely regarded as the most important feature in grading. The colorectal carcinoma has been graded as well differentiated (grade I), moderately differentiated (grade II), and poorly differentiated (grade III). In this study grade 2 was more common (69%) followed by grade 1 (18%) which correlated with the study done by Yalcin et al. [12]. In our study most of the tumors (50%) belong to pT3 followed by pT2 - (44%) and nodal metastasis was 36% in N1,12% in N2, and no nodal metastasis was seen in 52% - N0 which was in concordance with the study conducted by Abdou et al. [13]. When the comparison was made for stage, stage 1 and stage 3 predominated compared to stage 2 and stage 4. High stage was noted predominantly in males and more common in age group of >60 years.

In this study we studied the expression of VEGF in normal colonic epithelium, tubular adenoma, tubulovillous adenoma, villous adenoma, and carcinoma. 

In normal epithelium VEGF immunoreactivity was seen in all the 10 cases with a maximum intensity and a score of 300 which correlated with the study of Hanrahan et al. [14]. In his study, he found that there was an increase in VEGF expression in the adjacent tissue collected away from the primary tumor. In our study VEGF expression was seen both in adenomas and carcinomas.

In adenomas, VEGF expression was seen in 25 (64.1%) cases out of which VEGF expression was seen in 12 cases (60%) of tubular adenomas. The VEGF expression in tubulo villous adenoma was seen in nine cases (60%) and villous adenoma showed VEGF expression in all four cases (100%). There was a significant difference between the expression of VEGF in villous adenoma and tubular adenoma (p=0.05). The H score ranged from 40 to 300 in villous adenoma and 70 to 300 in tubular adenoma and in carcinomas expression was seen in 51% of cases.

From our study there was a slight increase in the expression of VEGF in colonic adenomas than the carcinomas with a mean of 216 in adenoma and 195 in carcinoma whereas in the study conducted by Abdou et al. [13], the VEGF expression was higher in carcinomas than in adenomas.

Molecular studies on transgenic mouse models have shown that angiogenesis begins in the premalignant phase of tumorogenesis, when dysplastic lesions acquire an increased microvasculature [15]. Thus this may correlate with our study where VEGF may be responsible for the induction of angiogenesis occurring in pre-malignant and early stages of CRC. 

In colorectal carcinomas, VEGF expression was seen in 18 cases (58.1%) <45 years of age and positive in eight cases (42.1%) >45 years. There was no significant difference in age with VEGF expression (p = 0.273). When VEGF expression was compared to the size, 11 cases (42.3%) were positive among tumors up to 5 cm and 15 cases (62.3%) were positive among tumors > 5 cm (p=0.153) which was statistically insignificant. Similar findings were observed by Abdou et al. [13] and Bendardaf et al. [16].

Tumor grade and stage in relation to VEGF positivity were analyzed for correlation. The VEGF was expressed high in grade 1 with a median score of 280 and a score of 210 in grade 2 and a median score of 180 in grade 3. Statistical data showed a p-value of 0.684. Conflicting studies were published considering VEGF expression and tumor differentiation. The VEGF expression was significantly more intense in poorly differentiated tumors in comparison with the well-differentiated ones in CRCs. In the present study, there was an inverse relationship between tumor grade and VEGF expression though it was insignificant. Similar findings were observed by Uner et al. [17] and Abdou et al. [13] although they did not reach a significant level.

In our study when the expression of VEGF and stage was compared a low expression was seen in advanced stage tumor. Stage 1 showed a maximum expression of 13 cases (65%), stage 2 showed an expression in four cases (44.4%), stage 3 (40%) in eight cases, and stage 4 in one case with a p-value of 0.303. According to Zheng et al. [18], Hanrahan et al. [14], and Uner et al. [17] no significant correlation between VEGF expression and stage was noticed which was similar to our study. Similar results were observed in esophageal and prostatic carcinoma. Berney et al. [19] found that VEGF expression was significantly reduced in metastatic colorectal carcinoma to the liver in comparison with the original primary tumor. These findings may support our results and explain the reduced VEGF expression with the advanced tumor stage observed in our study.

George et al. [20] and Saad et al. [21] observed a positive correlation between high VEGF expression and positive nodal status in CRC; in contrast, we observed a reversed association between VEGF expression and nodal status in CRC; this was in concordance with the study of Hanrahan et al. [14]. However, in our study as long as VEGF was highly expressed in superficial tumors (T1, T2), it is accepted to be over-expressed in tumors with negative nodal status as progression in the T stage is usually accompanied by progressive nodal involvement.

In view of the association of high VEGF expression in normal epithelium away from the tumor, adenomas, tumors of low grade, early stage, and negative nodal status by immune histochemical assessment, it could be suggested that VEGF may be an early carcinogenic factor in CRC. VEGF may be required for CRC development in early stages but as tumors become less differentiated, more aggressive, and deeply invading, it is no longer in need of VEGF that appears to be downregulated. So other angiogenic factors could have the upper hand in this stage of aggression and advancement of CRCs.

From our study reduced expression of VEGF in tumors with high grade and advanced stage necessitates further correlation with the molecular studies in CRCs in this part of India.

## Conclusions

From the above findings of our study, we conclude that VEGF could be considered as an early carcinogenic factor in colorectal carcinomas as it is expressed in higher intensity in precancerous lesions like adenoma and low-grade and stage 1 adenocarcinomas. Hence we infer that early colorectal carcinoma is an important model for targeted therapy with antiangiogenic factors for VEGF. The decreased expression of VEGF in higher grades and stages of adenocarcinomas suggests that it has a reduced role in tumor progression. Hence we suggest that the reduced role of VEGF in tumors with high grade and advanced stage necessitates further study of the molecular pathology of CRC in this part of India.
